# Unpacking the Black Box: Exploring Differences in Practices, Skills, and Knowledge Taught in School-Based Mindfulness Programs

**DOI:** 10.1007/s11121-025-01819-6

**Published:** 2025-06-25

**Authors:** Sebrina L. Doyle Fosco, Deborah L. Schussler

**Affiliations:** 1 Human Development and Family Studies Department, The Pennsylvania State University, University Park, PA 16802 USA; 2 Education Policy and Leadership Department, University at Albany, SUNY, Albany, NY 12222 USA

**Keywords:** School-based mindfulness curriculum, Core components, Education

## Abstract

School-based mindfulness programs (SBMPs) have shown a range of academic, emotional, and behavioral outcomes. However, heterogeneity in curricular content obscures accurate interpretation of results. To unpack the “black box” of SBMPs and better contextualize intervention outcomes, this investigation examined the practices, skills, and knowledge conveyed in twelve curricula. Required curricular elements (CEs) from the written curricula were coded for mindfulness practices and skills, and lessons were coded for mindfulness knowledge conveyed. Curricula had a different composition of components depending on theoretical foundation and program length. All curricula included intrapersonal (i.e., individual) practices, most often engaging somatic awareness. Interpersonal (i.e., relational) practices were identified in eight curricula and were infrequent, except in two programs that focused on kindness/compassion. All curricula cultivated intrapersonal skills of focused attention, emotion awareness, and emotion regulation at varying levels. Programs derived from MBSR cultivated the highest proportion of skills focused on awareness of mental states. Longer programs had a significantly higher proportion of CEs focused on interpersonal skills compared to shorter programs. Longer programs also had a higher proportion of lessons concentrated on focused attention and on empathy/perspective taking. Knowledge conveyed in lessons did not always correspond to the practices and skills coded, indicating students may be learning about topics even if they are not actively practicing or engaged in skill cultivation. This study offers clarity regarding the curricular content of SBMPs, making it possible to investigate links between active ingredients and program outcomes, refine theories of change, and better prepare teachers facilitating SBMPs.

**Supplementary Information:**

The online version contains supplementary material available at 10.1007/s11121-025-01819-6.

## Introduction

School-based mindfulness programs (SBMPs) are increasingly being recommended for prevention and treatment of mental health challenges and to promote wellbeing for youth in schools (DASH-CDC, 2023). This has only intensified as more evidence demonstrates strong links between students’ social, emotional, and behavioral development and academic learning (Cipriano et al., 2023). Research on individual SBMPs has shown myriad positive impacts; programs in the present study have shown promising intrapersonal effects on academic outcomes such as academic goal setting (Frazier et al., 2025), performance (Haines et al., 2023; Thierry et al., 2016), and engagement (Felver et al., 2014; Frank et al., 2017). Improvements to self-regulatory functioning included attention (Poehlmann-Tynan et al., 2016; Tolan et al., 2024), executive functioning (Crooks et al., 2020; Frank et al., 2021), emotion regulation (Fung et al., 2016; Schonert-Reichl et al., 2015), and behavior regulation (Felver et al., 2022). These SBMPs have also shown changes in mental health outcomes. Positive *intrapersonal* (i.e., individual) changes have included internalizing symptoms such as depression and anxiety (Bluth et al., 2016; Edwards et al., 2014; Frank et al., 2014). Positive *interpersonal* (i.e., relational) changes have included reductions in hostility and aggression (Felver et al., 2014; Frank et al., 2014; Matsuba et al., 2021) and increased prosociality and empathy (Frazier et al., 2025; Haines et al., 2023; Schonert-Reichl et al., 2015). However, results from two recent large-scale trials of the “.b” program showed no effects on socio-emotional or mental health outcomes (Kuyken et al., 2022); some students even reported poorer outcomes (Juul et al., 2025).

When accounting for null effects through meta-analyses and systematic reviews, the picture is more conservative. Meta-analyses on SBMPs for younger children have shown small effects on “attention, emotional and behavioral regulation, positive emotion and self-appraisal, and social competence and prosocial behavior” (Kander et al., 2024, p.1). Looking across developmental levels, small effects have been found on academic performance, impulsivity, and attention, but no differences were seen for interpersonal skills or student behavior (Mettler et al., 2023). Verhaeghen (2023), in addition to finding meta-analytic effects on academic performance, reported that longer interventions showed larger effects. Of note, these meta-analyses identified limitations to findings because of the methodological quality of the research design; for example, studies often had “business as usual” control groups instead of active controls.

Currently, there is a lack of clarity regarding SBMP content and implementation (Baelen et al., 2023; Roeser, Greenberg, et al., 2022a, 2022b). Content variation may be contributing to contradictory findings on effectiveness. Although programs use the common term SBMP, research has found “limited similarity between interventions” (Felver et al., 2023, p. 2), with each containing “a different, complex amalgamation of theories, aims, approaches, and techniques” (Semple et al., 2017, p. 48). Felver et al. even found disagreements between researchers and practitioners regarding what *should* constitute youth mindfulness-based program core components. Schussler et al. (2024) also found differences in curricular content of SBMPs by developmental level; specifically, less interpersonal mindfulness practices and skills (e.g., empathy, compassion) were evident in older grades.

There is also variation in who facilitates SBMPs. In published research, most SBMPs were led by trained mindfulness facilitators (Roeser et al., 2022a). These facilitators typically have personal and professional training and experience with mindfulness. Meanwhile, teachers implementing SBMPs are often provided training on the curriculum (Semple et al., 2017), but that training is often brief (e.g., 6 h; Maloney et al., 2016). Teacher facilitation has also been identified as a possible reason for the lack of intervention effects, especially when they are mindfulness-naive (Weare & Ormston, 2022). Moreover, with time and funds in short supply, schools may cut corners and only use resources absolutely required. Many SBMP curricula are available to freely download or purchase, so teachers may be asked to use the curricula without formal training. Therefore, the curriculum itself becomes both the way to teach students and the way to learn what one should be teaching.

This study examines “core curricular content,” which refers to mindfulness practices, skills, and knowledge articulated in SBMP written curricula. Consistent with SBMP reviews (e.g., Ergas & Hadar, 2019), a mindfulness practice is defined as a meditative act where a student is asked to take a specific orientation toward their experience, namely, “awareness that emerges through paying attention on purpose, in the present moment, and nonjudgmentally” (Kabat-Zinn, 2003, p.145). Mindfulness skills are dispositions, capacities, or habits cultivated through practicing mindfulness (Roeser, 2016). In Western society, mindfulness often focuses on individual, *intrapersonal* practices and skills involving awareness of and detachment from what is happening in the mind and body. Some propose there should be more focus on *interpersonal* practices and skills (Roeser et al., 2022b) that place attention and awareness on others and promote mindful and compassionate responding (Khoury et al., 2022). In this research, we examined core curricular content that is both intrapersonal and interpersonal.

Core curricular content is subsumed under the broader concept of “core components,” which also includes variables like characteristics of implementation, implementer, and setting (Blase & Fixsen, 2013; Dymnicki et al., 2020). Core components are the program elements “judged necessary to produce desired outcomes” and, therefore, “directly related to a program’s theory of change” (Blase & Fixsen, 2013, p.3). Theories related to the mechanisms of adult mindfulness programs exist (see Hölzel et al., 2011; Lindsay & Creswell, 2017); a recent systematic review of component studies (Stein & Witkiewitz, 2020) has defined and validated active ingredients producing outcomes in adult programs. Yet, adult needs differ from children’s. Lacking clear knowledge of core curricular content of SBMPs, it is difficult to formulate a theory of change delineating the essential program elements that produce intended outcomes. Exploring what is in the “black box” of these curricula is one way to answer the call for more research on core components of SBMPs (Baelen et al., 2023; Roeser et al., 2022a). The overarching research question for this study was: What would teachers teach if they used an SBMP curriculum exactly as written? Specifically, we examined the curriculum for (1) mindfulness practices presented, (2) mindfulness skills taught through practices and activities, and (3) mindfulness-related knowledge conveyed to students. As a post hoc exploratory analysis, we also examined how length of the curriculum related to content.

## Method

### Curriculum Selection

We identified mindfulness curricula, either researched in schools or marketed to teachers, available for download or purchase without proof of qualifications or paid training. Twelve SBMPs were used for these analyses (see Table 1). All SBMPs were identified as “mindfulness-based” (by the developer) and able to be used in PreK-12 schools, with a written curriculum available in English. Published research was not required for this exploratory, descriptive study, but a list of published program research is in Appendix A.

**Table 1:** Curricula used for content analysis

Curriculum	Req lessons	Total CEs (req CEs)	Grade	Published outcomes*	Foundation/focus
Dynamic Mindfulness (DM; Bose et al., 2016)	44	441 (441)	6–12	Yes	Movement
Pure Edge-Power (PE; Stern et al., 2016)	42	365 (348)	3–5^+^	Yes	Movement
Flourish (FLO; Harris et al., 2019)	37	374 (330)	4–5^+^	Yes	Movement
SEE Learning (SEE; Center for Contemplative Science & Compassion Based Ethics, 2019)	37	185 (171)	3–5^+^	Yes	Comp/Kind
Peace of Mind (POM; Ryden & Dodwell, 2016)	32	235 (219)	3–5^+^	No	Comp/Kind
Kindness (KIND; Healthy Minds Innovations, 2017)	24	161 (160)	PreK	Yes	Comp/Kind
Learning to BREATHE (L2B; Broderick, 2013)	18	116 (60)	6–12	Yes	MBSR
Wellness Works (WW; Kinder, 2017)	16	112 (96)	3–5^+^	No	SEL
MindUP (MU; The Hawn Foundation, 2011)	15	212 (76)	3–5^+^	Yes	SEL
MBSR-Teen (MBSRT; Beigel, 2018)	8	267 (169)	6–12	Yes	MBSR
Still Quiet Place (SQP; Saltzman, 2014)	8	105 (100)	All	No	MBSR
Soles of the Feet (SOTF; Felver & Singh, 2020)	5	69 (60)	All	Yes	Behavior

* peer-reviewed journals, +  = other versions available, *CEs* curricular elements, *req* required, *Comp/Kind* compassion/kindness, *SEL* social and emotional learning, *MBSR* Mindfulness-based stress reduction

### Procedure

We used a directed content analysis (Hsieh & Shannon, 2005) to examine curricular content of 12 SBMPs. Codes were primarily deductive and based on previous research on mechanisms of action for mindfulness, components posited for adult programs, and existing theories of change (Crane et al., 2017; Lawlor, 2016; Roeser, 2016; Stein & Witkiewitz, 2020). Some codes were also inductive, based on emergence in curricular content. The curriculum was coded exactly as written, rather than considering the myriad ways one might “enact” the curriculum (McCutcheon et al., 1988). All required lessons/CEs and homework were coded as if they had been completed as directed. Full implementation fidelity and access to all required material were assumed. Optional lessons/CEs were excluded given time barriers in education.

### Curriculum Coding

*Mindfulness Skills and Practices*. For skill and practice coding, lessons were unitized into curricular elements (CEs), denoted by different activities (e.g., didactic, practice, reflection) occurring throughout; when possible, the structure presented in the curriculum was followed (e.g., FLO has a developer-defined CE called “rest and reflection”). Occasionally, multiple practices/activities were under a single heading; in those cases, it was further divided.

For CEs that contained mindfulness practices, one code was awarded to capture the type of mindfulness practice (e.g., *mindful listening*). Practices were grouped into intrapersonal (i.e., *somatic practice, awareness of mental states, cultivating pleasant states, self-compassion/kindness*) and interpersonal categories (i.e., *mindful communication, empathy/perspective-taking, and compassion/kindness*; see Table 2). The codebook is available in Appendix B.

CEs with a mindfulness practice (e.g., awareness of emotions), discussion (e.g., discussing a story character’s emotions), class activity (e.g., emotion charades), or reflection (e.g., reflecting on one’s perspective) could be coded with up to two mindfulness skill codes (14.5% = 2 codes). No skill codes were awarded for purely didactic CEs (e.g., brain science). Both intrapersonal (i.e., *focused attention, open awareness, thought awareness, emotion awareness, emotion regulation, self-compassion/kindness*) and interpersonal skills were identified (i.e., *empathy/perspective taking, compassion/kindness, social connection*; see Table 3 and Appendix B).

*Knowledge–Lesson Topics Coding*. The lesson front matter (i.e., objective(s), purpose, goal(s), intention(s), and/or description) was coded for mindfulness-related knowledge using coding categories primarily aligned with mindfulness skills and practices. Two previous codes were expanded; the *Somatic* practice category was expanded to capture *somatic awareness* knowledge more broadly, and the *Social Connection* code was expanded to include the topic of *Interdependence*. Five additional codes emerged that were not directly seen as mindfulness but were identified across curricula, including *conflict resolution*, *neuroscience/brain science*, general *stress awareness/management*, *responsible decision-making*, and *embedding/extending* what was learned into “real life.” Each lesson received up to three knowledge codes (53.5% with two codes, 8.0% with three; see Table 4 and Appendix B).

*Reliability*. For skills/practices, three coders (including authors) coded one curriculum. Discrepancies were addressed, and the codebook was clarified. A second curriculum was double-coded to assure consistency. Individual coders coded the remaining curricula with ~ 22% of CEs purposefully selected for double coding; CEs were selected based on queries, which likely reduced reliability. Reliability was 73% based on percent agreement. For knowledge, two curricula were double-coded; across all codes, percent agreement was 93%. The codebook was clarified, and a third curriculum was double-coded; reliability was 97% based on percent agreement. Because of the high agreement, a single coder coded the remaining curricula with coding questions identified for discussion and rectification. All discrepancies and questions were rectified by consensus during regular team meetings. All coders had a personal mindfulness practice, and the coding team had expertise in mindfulness facilitation and K-12 education.

### Analyses

Coding and descriptive analyses were conducted in Excel. For all coding categories, a percentage was calculated for each SBMP that represented the total number of codes awarded for a given category divided by the total number of required CEs (skills/practices) or required lessons (knowledge). *T*-tests were also conducted in SPSS 28 to understand how length of the programs may have influenced content. SBMPs with 24 lessons or more were compared with those that were delivered in 18 lessons or less (i.e., less than half of the school year) to investigate relationships between curricular content and program length. Because this was exploratory and a small sample, we did not correct for the number of statistical tests; however, the more conservative *p*-value was used when Levene’s test for equality of variances was significant. All significant and marginally significant results are discussed.

## Results

### Intrapersonal Skills, Practices, and Knowledge

*Intrapersonal practices*. All SBMPs had intrapersonal mindfulness practices (range = 20.4%–73.5% of CEs), with the majority being somatic (range = 15.4%–60.4%). All programs included breathing practices, with DM having the highest proportion (range = 1.7%–18.8%). Eleven had Body Scan/Awareness practices with the highest proportion being in SOTF (range = 0.9%–36.7%). Ten curricula offered mindful movement/walking practices (range = 0.6%–21.7%); L2B, an MBSR-derived program, had the highest proportion. For sense practices, ten curricula had mindful listening (range = 0.6%–22.9%); WW and DM had the highest proportion. The remaining senses (see, smell, touch, taste) represented a small proportion of eight programs (range = 0.5%–9.0%) with MU and SQP having the greatest proportion.

Regarding non-somatic intrapersonal practices, *Awareness of Mental States* was coded in seven curricula (range = 0.9%–11.7%) with the MBSR-adapted curricula (L2B, MBSRT, SQP) having the greatest proportion. Practices for *Cultivating Pleasant States* were identified in nine curricula (range = 1.0%–19.7%); SEE, a compassion-based program which used resourcing/savoring as a core practice, had the highest proportion. Self-compassion practices were coded in low proportion in eight curricula (range = 0.5%–7.3%). No significant differences were found for intrapersonal practices by length of program (see Table 2 for more information).

**Table 2:** Percent of required curricular elements with mindfulness practice by program

	Longer						Shorter						Averages		
Practice	DM	PE	FLO	SEE	KIND	POM	L2B	WW	MU	MBSRT	SQP	SOTF	Total	Long	Short
***Intrapersonal***	***60.3%***	***38.5%***	***59.9%***	***40.0%***	***33.2%***	***20.4%***	***73.5%***	***67.7%***	***21.0%***	***25.5%***	***50.0%***	***48.3%***	***44.9%***	***42.1%***	***47.7%***
Somatic*	60.3%	25.0%	50.9%	18.0%	26.9%	17.1%	51.8%	60.4%	17.1%	15.4%	42.0%	48.3%	***36.1%***	***33.0%***	***39.2%***
* Breath*	*18.8%*	*5.5%*	*21.2%*	*11.8%*	*7.5%*	*13.9%*	*1.7%*	*18.8%*	*2.6%*	*5.9%*	*5.0%*	*11.7%*	***10.4%***	***13.1%***	***7.6%***
* Body Scan/Awareness*	*10.4%*	*0.9%*	*3.3%*	*1.1%*	*-*	*1.9%*	*5.0%*	*3.1%*	*1.3%*	*4.7%*	*2.0%*	*36.7%*	***5.9%***	***2.9%***	***8.8%***
* Movement/Walking*	*10.0%*	*13.8%*	*13.6%*	*0.6%*	*11.3%*	*-*	*21.7%*	*15.6%*	*2.6%*	*3.0%*	*9.0%*	*-*	***8.4%***	***8.2%***	***8.7%***
* Listening (senses)*	*20.6%*	*2.0%*	*11.8%*	*0.6%*	*6.9%*	*0.9%*	*1.7%*	*22.9%*	*5.3%*	*-*	*15.0%*	*-*	***7.3%***	***7.1%***	***7.5%***
* See/Smell/Touch/Taste*	*-*	*2.9%*	*0.9%*	*2.2%*	*1.2%*	*0.5%*	*-*	*-*	*5.2%*	*1.2%*	*9.0%*	*-*	***1.9%***	***1.3%***	***2.6%***
Awareness of Mental States	-	-	4.5%	1.7%	3.1%	0.9%	11.7%	-	-	6.5%	5.0%	-	***2.8%***	***1.7%***	***3.9%***
* Mental States*	*-*	*-*	*1.5%*	*0.6%*	*3.1%*	*-*	*1.7%*	*-*	*-*	*5.3%*	*4.0%*	*-*	***1.4%***	***0.9%***	***1.8%***
* Thoughts*	*-*	*-*	*-*	*1.1%*	*-*	*0.5%*	*5.0%*	*-*	*-*	*1.2%*	*1.0%*	*-*	***0.7%***	***0.3%***	***1.2%***
* Emotions*	*-*	*-*	*3.0%*	*-*	*-*	*0.5%*	*5.0%*	*-*	*-*	*-*	*-*	*-*	***0.7%***	***0.6%***	***0.8%***
Cultivating Pleasant States	-	12.9%	3.0%	19.7%	1.9%	1.9%	5.0%	-	3.9%	3.6%	1.0%	-	***4.4%***	***6.6%***	***2.3%***
* Gratitude*	*-*	*12.4%*	*1.8%*	*0.6%*	*1.9%*	*0.9%*	*5.0%*	*-*	*2.6%*	*1.8%*	*-*	*-*	***2.3%***	***2.9%***	***1.6%***
* Resourcing/Savoring*	*-*	*0.6%*	*1.2%*	*19.1%*	*-*	*0.9%*	*-*	*-*	*1.3%*	*1.8%*	*1.0%*	*-*	***2.2%***	***3.6%***	***0.7%***
Self-Compassion/Kindness	-	0.6%	1.5%	0.6%	1.3%	0.5%	5.0%	7.3%	-	-	2.0%	-	***1.6%***	***0.8%***	***2.4%***
* Positive Self-Talk*	*-*	*0.6%*	*-*	*0.6%*	*-*	*-*	*-*	*7.3%*	*-*	*-*	*-*	*-*	***0.7%***	***0.2%***	***1.2%***
* Self-Compassion/Kindness*	*-*	*-*	*1.5%*	*-*	*1.3%*	*0.5%*	*5.0%*	*-*	*-*	*-*	*2.0%*	*-*	***0.9%***	***0.6%***	***1.2%***
***Interpersonal***	***0.0%***	***2.6%***	***4.2%***	***2.8%***	***20.6%***	***38.0%***	***3.4%***	***0.0%***	***0.0%***	***2.4%***	***5.0%***	***0.0%***	***6.6%***	***11.4%***	***1.8%***
Compassion/Kindness	-	0.9%	3.0%	0.6%	18.1%	30.6%	1.7%	-	-	0.6%	2.0%	-	***4.8%***	***8.9%***	***0.7%***
Perspective Taking	-	-	0.3%	1.1%	-	-	-	-	-	-	1.0%	-	***0.2%***	***0.2%***	***0.2%***
Mindful Communication	-	1.7%	0.9%	1.1%	2.5%	7.4%	1.7%	-	-	1.8%	2.0%	-	***1.6%***	***2.3%***	***0.9%***
***Other Unspecified/Choice***	***0.0%***	***0.0%***	***0.0%***	***0.6%***	***0.0%***	***0.0%***	***5.0%***	***0.0%***	***0.0%***	***1.8%***	***1.0%***	***0.0%***	***0.7%***	***0.1%***	***1.3%***
***Not a practice***	***39.7%***	***58.9%***	***35.8%***	***56.7%***	***46.3%***	***41.7%***	***18.3%***	***32.3%***	***78.9%***	***70.4%***	***44.0%***	***51.7%***	***47.9%***	***46.5%***	***49.3%***

*Includes somatic practices where students were given a choice so it could not be categorized. - = 0%

*Intrapersonal skills. Focused Attention* (range = 27.6%–82.3%), *Emotion Regulation* (range = 2.1%–26.7%), and *Emotion Awareness* (range = 2.8%–20.0%) were cultivated in all SBMPs. WW was highest for CEs supporting the skill of *Focused Attention*, while SOTF represented the top of the range proportionally for *Emotion Regulation* and *Emotion Awareness*. *Thought Awareness* was identified in nine programs (range = 1.4%–15.0%) while *Open Awareness* was only coded infrequently in seven (range = 0.4%–5.0%); the MBSR-derived curricula were highest proportionally for both. Finally, *Self-compassion/Kindness* skills were coded in nine curricula (range = 1%–8%), with the highest proportion in SQP. No significant differences were found for intrapersonal skills by length (see Table 3).

**Table 3:** Percent of required curricular elements cultivating mindfulness skills by program

	Longer						Shorter						Averages		
Skill	DM	PE	FLO	SEE	POM	KIND	L2B	WW	MU	MBSRT	SQP	SOTF	Total	Long	Short
***Intrapersonal***	***69.2%***	***43.4%***	***68.8%***	***60.7%***	***44.4%***	***65.6%***	***90.0%***	***91.7%***	***34.2%***	***55.0%***	***78.0%***	***75.0%***	***64.7%***	***58.7%***	***70.7%***
Focused Attention	61.2%	33.6%	49.7%	41.6%	31.0%	45.6%	71.6%	82.3%	27.6%	28.4%	38.0%	46.7%	***46.4%***	***43.8%***	***49.1%***
Emotion Regulation	3.6%	3.8%	6.3%	25.3%	8.6%	3.1%	1.7%	2.1%	2.6%	4.2%	6.0%	26.7%	***7.8%***	***8.5%***	***7.2%***
Emotion Awareness	3.8%	7.1%	14.8%	2.8%	2.8%	17.5%	18.3%	5.2%	3.9%	14.3%	17.0%	20.0%	***10.6%***	***8.1%***	***13.1%***
Thought Awareness	4.1%	1.4%	2.4%	4.5%	1.4%	1.9%	10.0%	-	-	13.6%	15.0%	-	***4.5%***	***2.6%***	***6.4%***
Open Awareness	0.4%	-	0.6%	0.6%	0.5%	-	5.0%	-	-	3.0%	4.0%	-	***1.2%***	***0.4%***	***2.0%***
Self-Compassion/Kindness	-	2.8%	4.2%	4.5%	1.0%	3.7%	6.7%	2.1%	-	3.0%	8.0%	-	***3.0%***	***2.7%***	***3.3%***
***Interpersonal***	***1.1%***	***25.6%***	***23.9%***	***36.0%***	***50.5%***	***36.9%***	***5.0%***	***4.1%***	***18.4%***	***4.2%***	***5.0%***	***0.0%***	***17.6%***	29.0%*	***6.1%***
Compassion/Kindness	-	7.2%	6.0%	19.7%	36.1%	28.1%	3.3%	1.0%	9.2%	3.0%	3.0%	-	***9.7%***	***16.2%+***	***3.3%***
Empathy/Perspective Taking	0.2%	0.6%	9.1%	10.1%	3.7%	0.6%	-	-	7.9%	-	1.0%	-	***2.8%***	***4.1%***	***1.5%***
Social Connection	0.9%	18.4%	10.9%	14.7%	11.5%	25.6%	4.3%	3.1%	3.9%	1.2%	1.0%	-	***8.0%***	13.7%*	***2.3%***
***No code***	***30.4%***	***35.9%***	***15.5%***	***7.3%***	***16.2%***	***4.4%***	***8.3%***	***6.3%***	***48.7%***	***42.6%***	***20.0%***	***25.0%***	***21.7%***	***18.3%***	***25.2%***

14.5% of CEs were coded as cultivating more than one skill so total by SBMP can be more than 100%, * = *p* < .05, + = *p* < .10. - = 0%

*Intrapersonal knowledge*. Intrapersonal mindfulness lesson topics were present in all curricula (range = 46.9%–94.4%), most often through *Somatic Awareness* (range = 12.5%–80.0%). While all curricula cultivated the skill of *Focused Attention*, explicit learning objectives for *Focused Attention* that extended beyond attention to the body (coded as *Somatic Awareness* knowledge) were found in only six curricula (range = 5.1%–11.4%) with DM having the highest proportion. Although there was not a difference in *Focused Attention* skills or practices, longer programs had a significantly greater proportion of lessons that cultivated this knowledge (6.98% vs 1.11%; *t*(10) =  − 2.87, *p* = 0.017). In fact, all but one of the longer programs had lessons explicitly teaching *Focused Attention*, while only one of the shorter programs did.

Regarding other types of intrapersonal knowledge, *Emotion Regulation*, present as a skill in all curricula, was in lesson objectives/explanations for nine (range = 4.2%–50.0%); DM, SOTF, SQP, and WW had 20% or more lessons with this code. *Emotion Awareness* knowledge was cultivated in all programs (range = 3.1%–60.0%) with SOTF again having the highest proportion. *Thought Awareness* was coded in nine curricula (range = 4.8%–37.5%); the MBSR-derived curricula were highest (all over 20%). Eleven curricula had lesson topics *Cultivating Pleasant States* (range = 2.3%–20%) with MU and SOTF having the largest proportion. Finally, *Self-Compassion/Kindness* was coded in 10 curricula with L2B having the highest proportion (range = 2.6%–25%; see  4).

**Table 4:** Percent of required lessons focused on mindfulness and other topics by program

	Longer						Shorter						Averages		
Lesson Topic	DM	PE	FLO	SEE	POM	KIND	L2B	WW	MU	MBSRT	SQP	SOTF	Total	Longer	Shorter
***Intrapersonal***	***77.1%***	***66.7%***	***51.3%***	***62.2%***	***46.9%***	***75.0%***	***94.4%***	***81.3%***	***73.3%***	***50.0%***	***87.5%***	***80.0%***	***70.5%***	***63.2%***	***77.8%***
Somatic Awareness	50.0%	33.3%	17.9%	13.5%	18.8%	20.8%	44.4%	25.0%	46.7%	12.5%	12.5%	80.0%	***31.3%***	***25.7%***	***36.9%***
Focused Attention	11.4%	-	5.1%	10.8%	6.3%	8.3%	-	-	6.7%	-	-	-	***4.0%***	7.0%*	***1.1%***
Emotion Regulation	20.5%	9.5%	5.1%	8.1%	12.5%	4.2%	-	25.0%	-	-	50.0%	20.0%	***12.9%***	***10.0%***	***15.8%***
Emotion Awareness	15.9%	7.1%	18.0%	21.6%	3.1%	20.8%	22.2%	31.3%	13.3%	12.5%	25.0%	60.0%	***20.9%***	***14.4%***	***27.4%***
Thought Awareness	9.1%	4.8%	5.1%	8.1%	6.3%	-	22.2%	-	20.0%	25.0%	37.5%	-	***11.5%***	***5.6%***	***17.5%***
Cultivating Pleasant States	2.3%	11.9%	5.1%	16.2%	3.1%	16.7%	5.6%	-	20.0%	12.5%	12.5%	20.0%	***10.5%***	***9.2%***	***11.8%***
Self-Compassion/Kindness	9.1%	11.9%	2.6%	5.4%	3.1%	8.3%	22.2%	12.5%	-	12.5%	25.0%	-	***9.4%***	***6.7%***	***12.0%***
***Interpersonal***	***6.3%***	***21.4%***	***56.4%***	***48.6%***	***100.0%***	***37.5%***	***16.7%***	***0.0%***	***26.7%***	***12.5%***	***50.0%***	***0.0%***	***31.3%***	45.0%*	***17.6%***
Compassion/Kindness	2.3%	9.5%	15.4%	29.7%	96.9%	25.0%	11.1%	-	13.3%	-	25.0%	-	***19.0%***	***29.8%***	***8.2%***
Empathy/Perspective Taking	-	7.1%	25.6%	10.8%	9.4%	4.2%	-	-	6.7%	-	-	-	***5.3%***	9.5%*	***1.1%***
Mindful Communication	-	4.8%	7.7%	5.4%	3.1%	4.2%	-	-	6.7%	12.5%	12.5%	-	***4.7%***	***4.2%***	***5.3%***
Connection/Interdependence	4.5%	4.8%	15.4%	16.2%	3.1%	16.7%	5.6%	-	-	-	12.5%	-	***6.6%***	***10.1%+***	***3.0%***
***Other Topics***	***41.7%***	***38.1%***	***41.0%***	***16.2%***	***40.6%***	***12.5%***	***27.3%***	***18.8%***	***26.7%***	***62.5%***	***12.5%***	***20.0%***	***29.8%***	***31.7%***	***28.0%***
Stress Awareness/Management	20.5%	7.1%	7.7%	8.1%	-	-	11.1%	18.8%	6.7%	37.5%	-	-	***9.8%***	***7.2%***	***12.3%***
Responsible Decision Making	22.7%	9.5%	17.9%	8.1%	3.1%	4.2%	-	-	13.3%	25.0%	-	-	***8.7%***	***10.9%***	***6.4%***
Embedding/Extending	2.3%	-	2.6%	2.7%	6.3%	8.3%	16.7%	-	6.7%	12.5%	12.5%	20.0%	***7.5%***	***3.7%***	11.4%*
Conflict Resolution	-	7.1%	10.3%	-	25.0%	-	-	-	-	-	-	-	***3.5%***	***7.1%***	***0.0%***
Brain Science/Neuroscience	2.3%	14.3%	2.6%	-	9.4%	-	-	-	6.7%	-	-	-	***2.9%***	***4.7%***	***1.1%***

*p < .05, + = *p* < .10, - = 0%

### Interpersonal Skills, Practices, and Knowledge

*Interpersonal Practices*. Interpersonal practices were coded in eight curricula (range = 2.6%–38%), typically as *Compassion/Kindness* (range = 0.6%–30.6%). KIND and POM, which focused on compassion and kindness, had the highest proportion. *Empathy/Perspective Taking* practices were only found in three curricula SEE, FLO, and SQP (range = 0.3%–1.1%). *Mindful Communication* practices were used in eight curricula (range = 0.9%–7.4%); POM had the highest proportion (see Table 2). No significant differences were identified by length.

*Interpersonal Skills*. Interpersonal skills were coded in 11 curricula (range = 1.1%–50.5%). A significantly greater proportion of CEs were focused on *interpersonal skills* in the longer programs compared to shorter programs (29% vs. 6.12%; *t*(10) =  − 3.15, *p* = 0.01). *Compassion/Kindness* skills were identified in ten curricula (range = 1.0%–36.1%); there was also a marginally significant difference for these skills by program length indicating that longer programs proportionally had more (16.18% vs. 3.25%; *t*(5.51) =  − 2.19, *p* = 0.075). Indeed, SBMPs that identified a foundation of kindness/compassion (KIND, POM, SEE), all of which are longer programs, had the highest proportion. *Empathy/Perspective Taking* skills were identified in eight programs but infrequently (range = 0.2%–10.1%); SEE and FLO had the largest proportion. *Social Connection* skills were identified in all but one SBMP (range 0.9%–25.6%) with KIND having the highest proportion; these skills were also significantly proportionally higher in longer programs (13.67% vs. 2.25%; *t*(10) =  − 3.31, *p* = 0.008; see Table 3).

*Interpersonal Knowledge*. *Interpersonal Mindfulness* topics were identified in 10 curricula (range = 6.3% to 100%). *Compassion/Kindness* knowledge was identified in nine (range = 2.3%–96.9%); POM, with “Kindness Pals” in almost every lesson, had the highest proportion*.* Only six curricula had lessons focusing on *Empathy/Perspective Taking* (range = 4.2%–25.6%); longer programs had a significantly greater proportion (9.52% vs. 1.11%; *t*(10) =  − 2.24, *p* = 0.049). *Mindful Communication* was coded in eight curricula with MBSRT and SQP having the highest proportion (range = 3.1%–12.5%). Eight curricula also had lesson topics coded as *Social Connection/Interdependence* (range = 3.1%–16.7%). Longer programs had a marginally higher proportion of lessons addressing this topic (10.12% vs. 3.01%; *t*(10) =  − 2.08, *p* = 0.064); KIND and FLO had the highest proportion (see Table 4).

### Other Knowledge Topics

Other related topics were found in all curricula (range = 12.5%–41.7%). Eight had lessons focused on *Stress Awareness/Management* (range = 8.1% to 37.5%) and on *Responsible Decision-Making* (range = 3.1% to 25%). Ten curricula had lessons focusing on *Embedding/Extending* what was taught into daily life (range = 2.7%–20%); shorter programs had a significantly greater proportion of lessons focused on *embedding/extending* (11.39% vs. 3.69%; *t*(10) = 2.43, *p* = 0.036) possibly because they are not continuing these lessons for the majority of the school year in the same way as longer programs. Five curricula had lessons focused on *Brain Science/Neuroscience* (range = from 2.3% to 14.3%). Three curricula had lessons focused explicitly on *Conflict Resolution* (range = 7.1% to 25.0%; see Table 4).

## Discussion

This research aimed to unpack the black box of SBMPs, especially considering how teachers, novice to mindfulness, would use curricula. In this curricular content analysis for 12 SBMPs, the coding framework considered both intrapersonal and interpersonal mindfulness skills, practices, and knowledge. Previous research using this dataset identified developmental differences in skills and practices summarily across programs (Schussler et al., 2024). The current study adds to the discussion by clarifying the core curricular content of individual SBMPs and exploring variation based on length and theoretical foundation or focus.

Different amalgamations were found across these 12 curricula. Some programs were short and focused on teaching specific skills for specific issues (e.g., Soles of the Feet; Felver & Singh, 2020). Other longer programs like SEE Learning and Flourish were more holistic, covering a broader range of skills, practices, and knowledge. Movement-focused programs and compassion/kindness focused programs were typically longer, while MBSR-derived programs and programs focused on SEL were shorter. While program length is often talked about in terms of dosage or amount of the program received (e.g., Baelen et al., 2023), this research indicates that length of programs may also influence the content included.

In general, all SBMPs emphasized intrapersonal practices, skills, and knowledge. All programs included somatic practices, most often presenting breath awareness. Practices focusing on awareness of mental states, cultivation of pleasant states, and self-compassion were included less frequently and appeared somewhat dependent on theoretical foundations (e.g., MBSR-derived programs focused more on awareness of mental states). The intrapersonal skills of focused attention, emotion awareness, and emotion regulation were present in all curricula. Regarding intrapersonal knowledge, longer programs had a higher proportion of lessons cultivating knowledge of focused attention (e.g., a DM lesson objective states “*[Students will] build awareness that they have a choice where to focus attention*”). This difference may be partially explained by how this knowledge is discussed. While not significant, shorter programs had a greater proportion of lessons focused on *Somatic Awareness,* which may support building focused attention. Longer programs may allow for more abstract discussions.

Interpersonal practices were less frequent in SBMPs, with two exceptions, KIND and POM, which had core kindness/compassion practices featured across most lessons. Cultivation of interpersonal mindfulness skills was more prevalent than practices, given that skills could be taught through other activities, but these skills still were absent in some SBMPs. Generally, longer programs had a higher proportion of CEs focused on interpersonal mindfulness skills, especially *Social Connection* and *Compassion/Kindness*. Longer programs also had a higher proportion of lessons cultivating interpersonal mindfulness knowledge, specifically *Empathy/Perspective Taking* and *Social Connection/Interdependence*. Because interpersonal mindfulness requires the underpinnings of intrapersonal skills like emotion regulation (Khoury et al., 2022), shorter SBMPs may focus in on these topics while longer SBMPs have more time to include other aspects. Roeser et al., (2022b) have recently suggested that SBMPs need to move beyond the dualistic split between the intra- and interpersonal modes of teaching and incorporate both.

Including both intra- and interpersonal components may be important in relation to program outcomes. Research on adult interventions suggests that, while intrapersonal mindfulness impacts focused attention, interventions combining intra- and interpersonal components showed improvements across broader outcomes, including compassion, self-compassion, non-judgment, and empathic concern (Brito-Pons et al., 2018; Hildebrandt et al., 2017). Given the ubiquity of mindfulness in schools, and children’s unique developmental needs, an investigation of SBMPs may be warranted. This study provides the groundwork to explore what mindfulness practices, skills, and knowledge, taught through SBMP lessons and CEs, may be the “active ingredients” that are most robust in driving particular outcomes (S. Maloney et al., 2021; Stein & Witkiewitz, 2020).

An interesting finding was that lesson knowledge identified was sometimes different than the skills and practices in CEs; for example, in DM, less than 5% of CEs were identified with *Emotion Regulation* skills, but more than 20% of lessons focused on this topic. Some programs may rely more heavily on didactic instruction to convey knowledge about mindfulness skills. The effectiveness of different approaches warrants additional study. In this research, we only coded skills when students were actively engaged in learning. Research on effective academic instruction in areas like reading and math includes analyses of curricular modalities—i.e., knowledge vs skill development—and pedagogical processes—i.e., explicit instruction vs active learning (Mason & Otero, 2021; Stockard et al., 2018). Future analyses may benefit from exploring these differences to contextualize SBMP effectiveness and outcomes.

The results of this study echo what has been found in research elsewhere. For example, Felver et al. (2022) identified 22 potential core youth mindfulness program components, similar to those identified in this research, including mindful practices (e.g., somatic awareness, loving-kindness) and mindful skills (e.g., self-awareness, focused attention), as well as mindful attitudes/principles (e.g., non-judgment, non-reactivity); when asked to rank components, only nine were identified as “core” by at least 75% of researchers and mindfulness instructors. Even in a hypothetical space, there is disagreement on what these programs *should* contain. Further, Roeser et al., (2022a) identified heterogeneity in SBMP origins, length, facilitation, and outcomes, and Ergas and Hadar (2019) identified differences in pedagogies, aims, and framing. This research is valuable for prevention and intervention science because it highlights the importance of clarifying and contextualizing SBMPs, helping to fine-tune theories of change. Understanding what program developers included in these 12 youth-focused programs can help move the field closer to consensus on what constitutes an SBMP.

Attention to curricular content is also important for high-quality implementation of SBMPs. Most mindfulness content identified in these curricula may be difficult to teach with little or no training. Given that teachers may be asked to use an SBMP with only the curriculum to guide them, developers should consider an educative approach that accounts for the learning needs of students *and teachers*. Design principles include addressing adaptations for student needs (e.g., shortening lessons), providing representations of practice to support uptake (e.g., scripts), offering teacher knowledge support (e.g., student-friendly definitions of terms), and offering justifications for recommended practices (e.g., research available; Davis et al., 2017).

While it is recommended to provide SBMP curriculum training, teachers also need support for their own mindfulness so that they may embody and model these skills and practices with students (Frazier & Doyle Fosco, 2023; Schussler et al., 2022). The well-represented skills, practices and knowledge found across curricula in this study can be used to inform the content of this type of teacher training generally. Already, it has been suggested that teacher education should be augmented to include mindfulness instruction (e.g., Zimmerman, 2018).

### Limitations and Future Research

This study’s first limitation is the number of curricula represented. Because we were focused on what schools and districts could easily access, we did not include some well-known programs that required paid training. Additionally, although most programs did offer or recommend teacher training, we did not engage in the training because it was not required and beyond the project scope. We also did not review lower elementary curricula (K-2) due to overlap with grades 3–5. Further, we did not code for all aspects of mindfulness, as some required more inference and were, therefore, more difficult to code with reliability (e.g., mindful attitudes). Moreover, all required CEs were included in their entirety, which carries the assumption of 100% fidelity and active student engagement, rather than imposing real-world conditions. Finally, because most curricula did not specify an amount of time to spend on practices, proportions using CEs are only an approximation of the amount of the curricula that is concentrated on particular practices and skills. Some CEs would likely have taken longer than others but that is not captured in these analyses. Future research should address these limitations.

Additional research is needed to align the SBMP practices, skills, and knowledge with program outcomes. Meta-analytic and meta-regression strategies could help identify relationships between curricular components and important outcomes, similar to approaches taken for SEL (Cipriano et al., 2023). Identifying these high leverage curricular components (i.e., “program kernels”; Embry & Biglan, 2008; Jones et al., 2017) could support further refinement of teacher training content and help teachers intelligently adapt curricula to address time-related barriers, student needs, and contextual realities (e.g., Loucks et al., 2022).

## Supplementary Information

Below is the link to the electronic supplementary material.

ESM 1 (DOCX 28.5 KB)

ESM 2 (DOCX 19.6 KB)

## Data Availability

Data are available upon request from the first author.
