# Supplementary material for: Unpacking the Black Box: Exploring Differences in Practices, Skills, and Knowledge Taught in School-Based Mindfulness Programs
Source: Prev Sci. 2025 Jun 25;26(5):827–38. doi: 10.1007/s11121-025-01819-6 (PMC12245950; doi:10.1007/s11121-025-01819-6)
Supplement: Supplementary file 1 — (DOCX 28.5 KB) [file 11121_2025_1819_MOESM1_ESM.docx]

| *Appendix A. PK-12 Publications by Program* | |
| --- | --- |
| **Curriculum** | **Child/Adolescent outcomes reported** |
| **Kindness Curriculum**  **(**KIND; Healthy Minds Innovations, 2017) | **Increased:** academic performance, attention, emotion regulation, empathy, executive functioning, prosociality, self-regulation |
| 1. Flook, L., Goldberg, S. B., Pinger, L., & Davidson, R. J. (2015). Promoting prosocial behavior and self-regulatory skills in preschool children through a mindfulness-based Kindness Curriculum. Developmental Psychology, 51(1), 44–51. <https://doi.org/10.1037/a0038256> 2. Haines, B. A., Hong, P. Y., Immel, K. R., & Lishner, D. A. (2023). The mindfulness-based Kindness Curriculum for preschoolers: An applied multi-site randomized control trial. Mindfulness, 14(9), 2195–2210. <https://doi.org/10.1007/s12671-023-02210-8> 3. Poehlmann-Tynan, J., Vigna, A. B., Weymouth, L. A., Gerstein, E. D., Burnson, C., Zabransky, M., Lee, P., & Zahn-Waxler, C. (2016). A pilot study of contemplative practices with economically disadvantaged preschoolers: Children’s empathic and self-regulatory behaviors. Mindfulness, 7(1), 46–58. <https://doi.org/10.1007/s12671-015-0426-3> | |
| **Social, Emotional & Ethical (SEE) Learning**  (SEE; Center for Contemplative Science & Compassion Based Ethics, 2019) | **Increased:** empathy, goal setting, prosociality, self-compassion |
| 1. Frazier, T., Roeser, R. W., Schonert-Reichl, K. A., & Tenzin Negi, L. (2025). Compassion training in elementary school children. PLoS ONE, online, hal-04734160. https://hal.science/hal-04734160v1 | |
| **Peace of Mind***  (POM; Ryden & Dodwell, 2016) | **Increased:** behavioral and emotional regulation |
| 1. Fegans Gould, L. (2018). Peace of Mind pilot study results 2018: executive summary. <https://teachpeaceofmind.org/evaluation/> | |
| **MindUP**  (MU; The Hawn Foundation, 2011) | **Increased:** academic performance, adaptive skills, attention, cognitive and emotional control, empathy, executive functioning, happiness, mindfulness, optimism, prosociality, stress management, self-compassion, self-regulation  **Decreased:** aggression, behavior problems, emotion suppression, internalizing, negative emotion |
| 1. Crooks, C. V, Bax, K., Delaney, A., Kim, H., & Shokoohi, M. (2020). Impact of MindUP among young children: Improvements in behavioral problems, adaptive skills, and executive functioning. Mindfulness, 11, 2433–2444. <https://doi.org/10.1007/s12671-020-01460-0> 2. de Carvalho, J. S., Pinto, A. M., & Marôco, J. (2017). Results of a mindfulness-based social-emotional learning program on Portuguese elementary students and teachers: A quasi-experimental study. Mindfulness, 8(2), 337–350. <https://doi.org/10.1007/s12671-016-0603-z> 3. Schonert-Reichl, K. A., & Lawlor, M. S. (2010). The effects of a mindfulness-based education program on pre- and early adolescents’ well-being and social and emotional competence. Mindfulness, 1(3), 137–151. <https://doi.org/10.1007/s12671-010-0011-8> 4. Schonert-Reichl, K. A., Oberle, E., Lawlor, M. S., Abbott, D., Thomson, K., Oberlander, T. F., & Diamond, A. (2015). Enhancing cognitive and social – emotional development through a simple-to-administer mindfulness-based school program for elementary school children: A randomized controlled trial. Developmental Psychology, 51(1), 52–66. <https://doi.org/10.1037/a0038454> 5. Thierry, K. L., Bryant, H. L., Nobles, S. S., & Norris, K. S. (2016). Two-year impact of a mindfulness-based program on preschoolers’ self-regulation and academic performance. Early Education and Development, 27(6), 805–821. <https://doi.org/10.1080/10409289.2016.1141616> | |
| **Wellness Works***  (WW; Kinder, 2017) | **Increased:** executive functioning |
| 1. Desmond, C. T., Kinder, W., Hanich, L. B., & Chukwu, O. C. B. (2019). Wellness Works in schools: The practice and research of a mindfulness program in urban middle schools. In I. Ivtzan (Ed.), Handbook of mindfulness-based programmes: Mindfulness interventions from education to health and therapy (1st ed., pp. 231–240). Routledge. | |
| **Flourish Curriculum**  (FLO; Harris et al., 2019) | **Increased:** attention, prosociality, self-efficacy  **Decreased:** conduct problems for students in high poverty schools |
| 1. Tolan, P. H., Harris, A. R., Burchinal, M., & Jennings, P. A. (2024). Promoting 21st century health and wellness skills in elementary school children: a group randomized trial. Prevention Science, 0123456789. https://doi.org/10.1007/s11121-024-01717-3 | |
| **Pure Edge- Power**  (PE; Stern et al., 2016) | **Increased:** academic performance, sleep, self-esteem, self-regulation  **Decreased:** stress |
| 1. Chick, C. F., Singh, A., Anker, L. A., Buck, C., Kawai, M., Gould, C., Cotto, I., Schneider, L., Linkovski, O., Karna, R., Pirog, S., Parker-Fong, K., Nolan, C. R., Shinsky, D. N., Hiteshi, P. N., Leyva, O., Flores, B., Matlow, R., Bradley, T., … O’Hara, R. (2022). A school-based health and mindfulness curriculum improves children’s objectively measured sleep: a prospective observational cohort study. Journal of Clinical Sleep Medicine, 18(9), 2261–2271. <https://doi.org/10.5664/jcsm.9508> 2. Wang, D., & Hagins, M. (2016). Perceived benefits of yoga among urban school students: A qualitative analysis. <https://doi.org/10.1155/2016/8725654> | |
| **Learning to BREATHE**  (L2B; Broderick, 2013) | **Increased:** attention, calmness, emotion regulation, resilience, self-acceptance, self-efficacy, self-esteem  **Decreased:** behavior problems, emotion suppression internalizing, perceived stress, rumination, somatic symptoms, tiredness |
| 1. Bluth, K., Campo, R. A., Pruteanu-Malinici, S., Reams, A., Mullarkey, M., & Broderick, P. C. (2016). A school-based mindfulness pilot study for ethnically diverse at-risk adolescents. Mindfulness, 7(1), 90–104. <https://doi.org/10.1007/s12671-014-0376-1> 2. Broderick, P. C. (2009). Learning to BREATHE: A pilot study of a mindfulness curriculum for adolescents. Advances in School Mental Health Promotion, 2(1), 35–46. 3. Eva, A. L., & Thayer, N. M. (2017). Learning to BREATHE: A pilot study of a mindfulness-based intervention to support marginalized youth. Journal of Evidence-Based Complementary and Alternative Medicine, 22(4), 580–591. <https://doi.org/10.1177/2156587217696928> 4. Felver, J. C., Clawson, A. J., Morton, M. L., Brier-Kennedy, E., Janack, P., & DiFlorio, R. A. (2019). School-based mindfulness intervention supports adolescent resiliency: A randomized controlled pilot study. International Journal of School and Educational Psychology, 7(sup1), 111–122. <https://doi.org/10.1080/21683603.2018.1461722> 5. Fung, J., Guo, S., Jin, J., Bear, L., & Lau, A. (2016). A pilot randomized trial evaluating a school-based mindfulness intervention for ethnic minority youth. Mindfulness, 7(4), 819–828. <https://doi.org/10.1007/s12671-016-0519-7> 6. Fung, J., Kim, J. J., Jin, J., Chen, G., Bear, L., & Lau, A. S. (2019). A randomized trial evaluating a school-based mindfulness intervention for ethnic minority youth: Exploring mediators and moderators of intervention effects. Journal of Abnormal Child Psychology, 47(1), 1–19. <https://doi.org/10.1007/s10802-018-0425-7> 7. Metz, S. M., Frank, J. L., Reibel, D., Cantrell, T., Sanders, R., & Broderick, P. C. (2013). The Effectiveness of the Learning to BREATHE Program on Adolescent Emotion Regulation. Research in Human Development, 10(3), 252–272. <https://doi.org/10.1080/15427609.2013.818488> | |
| **Dynamic Mindfulness**  (DM; Bose et al., 2016) | **Increased:** coping skills, emotion regulation, school engagement, self-control  **Decreased:** hostility, internalizing, intrusive thoughts, rumination, physical and emotional arousal, perceived stress |
| 1. Frank, J. L., Bose, B., & Schrobenhauser-Clonan, A. (2014). Effectiveness of a school-based yoga program on adolescent mental health, stress coping strategies, and attitudes toward violence: Findings from a high-risk sample. Journal of Applied School Psychology, 30(1), 29–49. <https://doi.org/10.1080/15377903.2013.863259> 2. Frank, J. L., Kohler, K., Peal, A., & Bose, B. (2017). Effectiveness of a school-based yoga program on adolescent mental health and school performance: Findings from a randomized controlled trial. Mindfulness, 8(3), 544–553. <https://doi.org/10.1007/s12671-016-0628-3> 3. Ramadoss, R., & Bose, B. (2010). Transformative life skills: Pilot study of a yoga model for reduced stress and improving self-control in vulnerable youth. International Journal of Yoga Therapy, 20(1), 73–78. <https://doi.org/10.17761/ijyt.20.1.a4214885w7101046> | |
| **MBSR-Teen**  (MBSRT; Beigel, 2018) | **Increased:** mindfulness, self-compassion  **Decreased:** depression, perceived stress |
| 1. Edwards, M., Adams, E. M., Waldo, M., Hadfield, O. D., & Biegel, G. M. (2014). Effects of a mindfulness group on Latino adolescent students: Examining levels of perceived stress, mindfulness, self-compassion, and psychological symptoms. Journal for Specialists in Group Work, 39(2), 145–163. <https://doi.org/10.1080/01933922.2014.891683> | |
| **Soles of the Feet**  (SOTF; Felver & Singh, 2020) | **Increased:** academic engagement, attention regulation **Decreased:** aggression, off-task/disruptive behavior |
| 1. Felver, J. C., Clawson, A. J., Ash, T. L., Martens, B. K., Wang, Q., & Singh, N. N. (2022). Meta-analysis of mindfulness-based program Soles of the Feet for disruptive behaviors. Behavior Modification. <https://doi.org/10.1177/01454455211073738> 2. Felver, J. C., Felver, S. L., Margolis, K. L., Kathryn Ravitch, N., Romer, N., & Horner, R. H. (2017). Effectiveness and social validity of the Soles of the Feet mindfulness-based intervention with special education students. Contemporary School Psychology, 21(4), 358–368. <https://doi.org/10.1007/s40688-017-0133-2> 3. Felver, J. C., Tipsord, J. M., Morris, M. J., Hyatt Racer, K., & Dishion, T. J. (2017). The Effects of mindfulness-based intervention on children’s attention regulation. Journal of Attention Disorders, 21(10), 872–881. <https://doi.org/1087054714548032> 4. Singh, N. N., Lancioni, G. E., Manikam, R., Winton, A. S. W., Singh, A. N. A., Singh, J., & Singh, A. D. A. (2011). A mindfulness-based strategy for self-management of agressive behavior in adolescents with autism. Research in Autism Spectrum Disorders, 5, 1153–1158. <https://doi.org/10.1016/j.rasd.2010.12.012> 5. Singh, N. N., Lancioni, G. E., Myers, R. E., Karazsia, B. T., Courtney, T. M., & Nugent, K. (2017). A mindfulness-based intervention for self-management of verbal and physical aggression by adolescents with Prader–Willi syndrome. Developmental Neurorehabilitation, 20(5), 253–261. <https://doi.org//dx.doi.org/10.3109/17518423.2016.1141436> | |
| **Still Quiet Place***  (SQP; Saltzman, 2014) | **Increased:** attention, self-compassion  **Decreased:** negative emotion, self-judgment |
| 1. Saltzman, A., & Goldin, P. (2008). Mindfulness-based stress reduction for school-age children. In L. A. Greico & S. C. Hayes (Eds.), Acceptance and mindfulness treatments for children and adolescents: A practitioners guide (pp. 139–161). New Harbinger Publications. | |
| *Note: * = Peer-reviewed journal articles not available. Drawing from best sources available.* | |
