# Supplementary material for: Unpacking the Black Box: Exploring Differences in Practices, Skills, and Knowledge Taught in School-Based Mindfulness Programs
Source: Prev Sci. 2025 Jun 25;26(5):827–38. doi: 10.1007/s11121-025-01819-6 (PMC12245950; doi:10.1007/s11121-025-01819-6)
Supplement: Supplementary file 2 — (DOCX 19.6 KB) [file 11121_2025_1819_MOESM2_ESM.docx]

**Appendix B. Codebook**

**SBMP mindful practices**

| **MF Practice** | **Definition** |
| --- | --- |
| ***Intrapersonal practices*** | |
| **Somatic Practices** | **Awareness of the body** |
| Unspecified | General practice involving the body. |
| MF Breathing | Any activity primarily focused on breathing. |
| Body Scan/Awareness | Bringing attention to specific parts of the body while being still. |
| Mindful Movement | Bringing attention to the body while it is moving. |
| Mindful Walking | Bringing attention to walking. |
| Mindful Touch | Awareness of something one is touching with a part of the body. |
| MF Smelling | Bringing attention to the olfactory sense. |
| Mindful Seeing | Bringing attention to something one is looking at. |
| Mindful Eating/Tasting | Bringing attention to something one is eating. |
| Mindful Listening | Bringing attention to the sounds one is hearing. |
| **Awareness of Mental States** | **Awareness of thoughts/emotions** |
| Aware of Mental States | Awareness of both one’s thoughts and emotions. |
| Aware of Thoughts | Awareness of one’s thoughts. |
| Aware of Emotions | Awareness of one’s feelings. |
| **Cultivating Pleasant Experiences** | **Appreciation for the present and the future** |
| Resourcing/Savoring | Allowing oneself to linger on a thought, feeling, sensation that brings wellbeing. |
| Gratitude | Cultivating appreciation. |
| **Self-Kindness/Compassion** | **Being kind to oneself** |
| Positive Self-Talk | Cultivating an inner voice of acceptance and non-judgement. |
| Self-Compassion | Directing kindness toward the self. |
| ***Interpersonal Mindfulness*** | |
| Mindful Communication | Listening to understand others from their perspective; Speaking clearly. |
| Empathy/Perspective Taking | Understanding how another thinks or feels in situations. |
| Compassion/Kindness | Taking action to meet the needs of another. |

**SBMP mindful skills**

| **MF Skill** | **Definition** |
| --- | --- |
| ***Intrapersonal Mindfulness*** | |
| Emotion Regulation | The process of modulating one or more aspects of an emotional experience or response. Assumed to refer to both subjective experience and emotion-related behavioral responses (Chambers et al., 2009). |
| Focused Attention | Direct and sustained attention on a select object with ability to nonjudgmentally detect and disengage from mind wandering and distractors (Lutz et al., 2008). |
| Open Awareness | Without an explicit object of attention, the non-reactive, metacognitive awareness and monitoring of cognitive/emotional interpretations of stimuli (Lutz et al., 2008). *An anchor could be offered during the practice.* |
| Emotion Awareness | Attending to and understanding one's own emotions (Boden & Thompson, 2015). This includes awareness of pleasant, unpleasant, and neutral feelings. |
| Thought Awareness | Capacity to consciously and intentionally turn the focus of thought onto the thought itself; an “explicit characterization of what is currently being experienced” (Schooler et al, 2015) |
| Self-Kindness/Self-Compassion | a) Being kind and understanding toward oneself, b) Perceiving one's own suffering as part of a larger human experience, c) Holding painful feelings and thoughts in mindful awareness (Neff) |
| ***Interpersonal Mindfulness*** | |
| Empathy | The capacity to comprehend or experience the emotions of another (Hogan, 1969 and Bryant, 1982 as cited in Jolliffee & Farrington, 2006). |
| Social Connection | A person’s subjective sense of having close and positively experienced relationships with others in the social world (Seppala et al., 2013). |
| Compassion/Kindness | The recognition of suffering and the desire to help one who is suffering (compassion) (Goetz et al., 2010).  Wish for all to experience happiness (kindness) (Shonin et al 2015). |

**SBMP Lesson Objectives**

| **Objective** | **Definition** |
| --- | --- |
| ***Intrapersonal Mindfulness*** | |
| Focused attention | Addresses focused attention not otherwise covered in another category (e.g., if already coding somatic awareness, focused attention is assumed). |
| Somatic Awareness | Awareness of the body |
| Thought Awareness | Awareness of one’s thoughts. |
| Emotion Awareness | Awareness of one’s feelings/emotions. |
| Emotion Regulation | Regulation of one's emotions. |
| Self-Kindness/Compassion | Being kind/compassionate to one's self including use of positive self-talk strategies |
| Cultivating Pleasant Experiences/States | Appreciation for the present and/or the future though activities including resourcing/savoring, gratitude, optimism. |
| ***Interpersonal Mindfulness*** | |
| Perspective taking/Empathy | Understanding how another thinks or feels in situations. |
| Compassion/Kindness | Taking action to meet the needs of another. *can include non-human beings/entities such as the earth, insects, trees |
| Social Connection/ Interdependence | Including social connection, connection with the environment, a feeling of connection with your community, the world |
| Mindful Communication | Listening to understand others; Speaking clearly. |
| ***Other Topics*** | |
| Conflict Resolution | Steps to address or reduce conflict with others |
| Brain science/neuroscience | Anything relating to teaching on areas of the brain or links between brain and body, or functions of the brain (e.g., negativity bias) |
| Responsible Decision Making | The abilities to make caring and constructive choices about personal behavior and social interactions across diverse situations (CASEL). This can include intention setting, goal setting, making healthy choices, leadership behaviors, and taking/understanding mindful or right action. |
| Stress Awareness/Management | Focused on stress management broadly defined and not otherwise addressed with other topics |
| Embedding/extending | Discussion around what is next, how to use mindful practices in life |
